# Pleomorphic Carcinoma With *EGFR* and Concomitant Mutations Transformed From Lung Adenocarcinoma: A Case Report

**DOI:** 10.1002/cnr2.70546

**Published:** 2026-04-19

**Authors:** Yuki Akazawa, Takashi Sato, Yuri Yagami, Yurika Kesen, Tomohiro Furo, Atsuko Watanabe, Jumpei Endo, Maito Nakano, Hiroto Kurata, Hiromi Matsuo, Yoshiro Nakahara, Jiichiro Sasaki, Katsuhiko Naoki

**Affiliations:** ^1^ Department of Respiratory Medicine Kitasato University School of Medicine Sagamihara Japan; ^2^ Department of Pathology Kitasato University School of Medicine Sagamihara Japan; ^3^ Research and Development Center for New Medical Frontiers Kitasato University School of Medicine Sagamihara Japan

**Keywords:** drug resistance, *EGFR* mutation, genomic profiling, histological transformation, lung cancer, pleomorphic carcinoma

## Abstract

**Background:**

Transformation to small cell carcinoma, squamous cell carcinoma, and rarely, pleomorphic carcinoma (PC) has been reported to be found in *EGFR*‐mutated lung cancer with acquired resistance to EGFR tyrosine kinase inhibitors (TKIs). However, evidence for underlying factors including genomic profiles associated with PC transformation has been lacking.

**Case:**

We report a case of *EGFR*‐mutated lung adenocarcinoma that transformed to PC confirmed by an autopsy after stereotactic radiotherapy and treatment with EGFR‐TKIs. Comprehensive genomic profiling of the autopsy tissue revealed *BRAF* G466A, *KRAS* L19F, and *NF1* Q2324* mutations in addition to *EGFR* E746_A750del. Anti‐cancer treatments with radiation and EGFR‐TKIs accompanying these gene alterations might induce the histological transformation and resistance to EGFR‐TKIs.

**Conclusion:**

This report adds an insight into the involvement of genetic events in histological transformation in *EGFR*‐mutated lung cancer.

## 1 Introduction

The use of EGFR tyrosine kinase inhibitors (EGFR‐TKIs) has substantially improved the clinical outcomes of patients with non‐small cell lung cancer harboring activating *EGFR* mutations; however, most tumors eventually relapse after treatment with EGFR‐TKIs. Various studies have reported resistance mechanisms against the drugs, such as on‐target resistance due to alterations of *EGFR* itself and off‐target resistance through bypass signaling pathways [1]. Trans‐differentiation to other histological subtypes such as small cell carcinoma, squamous cell carcinoma, and rarely, pleomorphic carcinoma (PC) has also been reported to be related to the resistance mechanisms [2, 3]. While genomic alterations in small cell carcinoma and squamous cell carcinoma transformed from lung adenocarcinoma have been explored by analyzing clinical specimens of the transformed tumors [4, 5], evidence on those in transformed pulmonary PC has been lacking.

Here, we present a case of *EGFR*‐mutated lung cancer that eventually transformed to PC after stereotactic radiotherapy and treatment with EGFR‐TKIs. Comprehensive genomic profiling of the autopsy tissue identified concomitant mutations in *BRAF*, *KRAS*, and *NF1* in addition to the *EGFR* mutation. The anti‐cancer treatments and these concomitant genetic events might be associated with the histological transformation and resistance to EGFR‐TKIs.

## 2 Case Presentation

**FIGURE 1:**
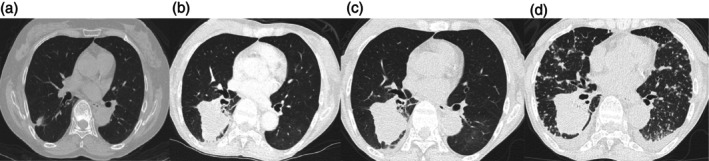
Computed tomography scans at initial diagnosis (a), at the time of recurrence after 11 years (b), after 3 months of osimertinib treatment (c), and at 3 months after osimertinib discontinuation (d).

**FIGURE 2:**
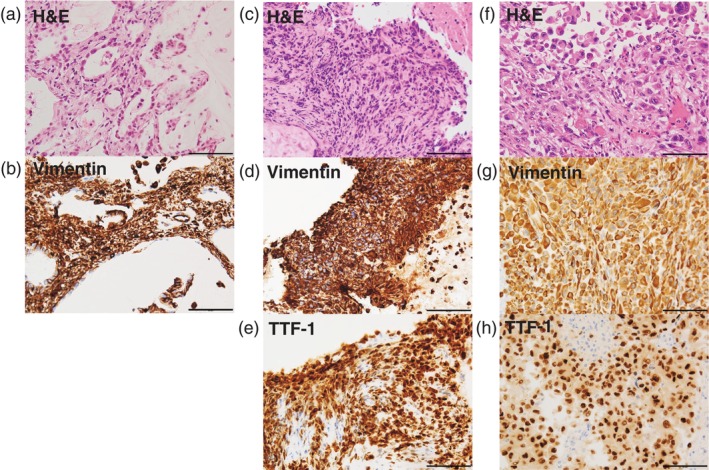
Histopathological findings from transbronchial biopsy at initial diagnosis (a, b) and at the time of recurrence after 11 years (c–e), and from autopsy (f–h). Original magnification, 200×. Scale bar, 100 μm.

**FIGURE 3:**
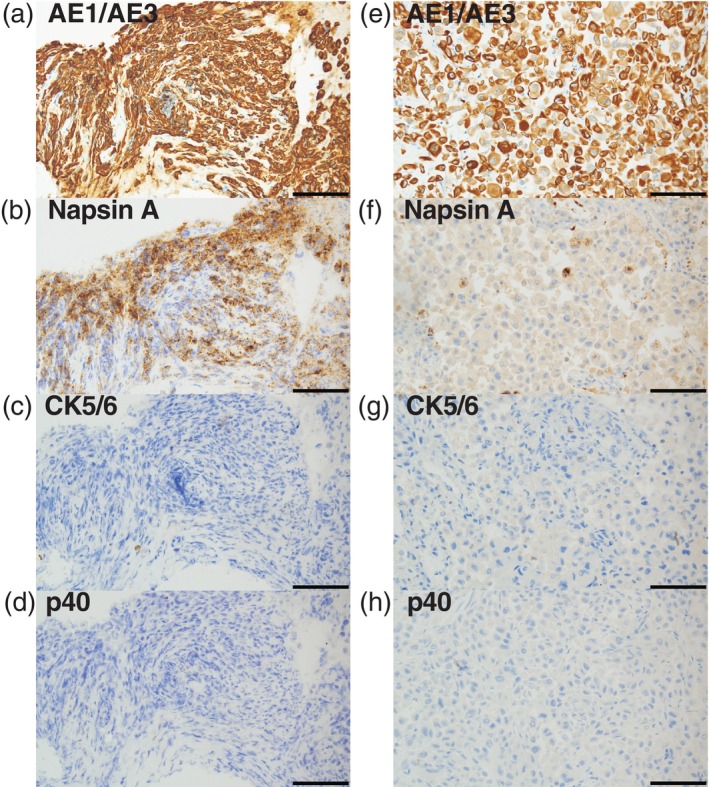
Additional immunohistochemical staining of transbronchial biopsy at the time of recurrence (a–d), and autopsy (e–h). Original magnification, 200×. Scale bar, 100 μm.

A 72‐year‐old Japanese female never smoker with the ECOG performance status of 1 presented to our department (Sagamihara, Japan) in 2010 with an asymptomatic lung nodule in the right lower lobe. She had a medical history of rheumatoid arthritis which had been treated with methotrexate and prednisolone, and no family history. She was diagnosed with clinical stage IA adenocarcinoma (Figures 1a and 2a,b) and received stereotactic body radiation therapy (SBRT). Eleven years later in 2021, she developed a local recurrence in the same lobe of the right lung with bone metastases (Figure 1b). A transbronchial biopsy suggested that the tumor histology was adenocarcinoma (Figures 2c–e and 3a–d), and the subsequent molecular analyses of the tissue detected *EGFR* exon 19 deletion and the programmed death‐ligand 1 expression of 80% (according to the tumor proportion score). She started osimertinib, an EGFR‐TKI, and then the tumor kept stable in size (Figure 1c). However, 3 months later from the start, she discontinued osimertinib due to cellulitis in both lower legs, and another 3 months after the discontinuation, the disease progressed in the primary lesion and mediastinal lymph nodes with the appearance of multiple lung metastases and liver metastasis (Figure 1d). Meanwhile, her activities of daily living declined, and the performance status became 3. Although gefitinib was started, her respiratory condition worsened due to the disease progression, and the patient succumbed to the disease shortly.

A pathological autopsy revealed PC of the lung with metastatic lesions in the lungs, liver, right adrenal gland, right kidney, pancreas, thyroid, stomach, right pleura, pericardium, mesentery, brain and mediastinal lymph nodes (Figure 2f–h). Immunohistochemical staining of the biopsy and autopsy specimens (Figures 2, 3) showed that these cancer tissues were originally positive for vimentin in addition to thyroid transcription factor‐1 (Figure 2b,d,e,g,h). On the other hand, although the biopsy cancer tissues were positive for Napsin A (Figure 3b), the autopsy tissues were only focally positive for it (Figure 3f). To investigate the underlying genetic features of this transformed cancer, we performed comprehensive genomic profiling of the autopsy tissue by Azenta Pan‐Cancer Panel and it revealed *BRAF* G466A, *KRAS* L19F, and *NF1* Q2324* mutations in addition to *EGFR* E746_A750del in the tumor cells (Table 1).

**TABLE 1:** Gene alterations identified by comprehensive genomic profiling of the autopsy tissue.

Gene name	Ref > alt	Chr: Position	Effect	Amino acid change
*EGFR*	AGGAATTAAGAGAAGC>A	7:55174771	In‐frame deletion	E746_A750del
*BRAF*	C>G	7:140781611	Missense	G466A
*KRAS*	C>G	12:25245328	Missense	L19F
*NF1*	C>T	17:31340553	Nonsense	Q2324*

## 3 Discussion

In the present case of *EGFR*‐mutated lung cancer, pathological and genomic assessment of the autopsy specimens allowed us to reveal PC transformation with the concomitant oncogenic mutations. To the best of our knowledge, evidence for genomic events associated with PC transformation of *EGFR*‐mutated lung cancer has been lacking. Therefore, we highlight the implication of the concomitant gene alterations relevant to PC transformation and the possible usefulness of comprehensive genomic profiling in the investigation of trans‐differentiation as a resistance mechanism to EGFR‐TKIs.

In this case, the tumor did not shrink after osimertinib was started and later gefitinib could not stop it from progression. It remains unclear whether the tumor finally transformed from adenocarcinoma to PC after treatment with EGFR‐TKIs, or it was already pleomorphic carcinoma in part when the tumor first relapsed 11 years after stereotactic radiotherapy, since the biopsy tissue was small and crushed. Additionally, positive staining of vimentin in the tumor cells suggests the original mesenchymal differentiation of this tumor. Nevertheless, the differentiation state of PC can be a possible resistance mechanism to the EGFR‐TKIs. Although radiation‐induced sarcomas have been reported in various organs [6], to our knowledge, pulmonary PC associated with radiation has not been reported. On the other hand, there have been several reports of histological transformation to pleomorphic carcinoma following treatment with EGFR‐TKIs (Table 2) [7, 8, 9, 10, 11]. In a broader sense than PC transformation, epithelial to mesenchymal transition (EMT) has been reported as one of the resistance phenotypes in *EGFR*‐mutated lung cancers resistant to the TKIs, accounting for 1%–2% of them [12, 13, 14], and the resistance mechanisms mediated by EMT have been further explored [15, 16, 17].

**TABLE 2:** Summary of previous cases of PC transformation after EGFR‐TKIs in *EGFR*‐mutated lung cancer.

Authors	Initial histology, *EGFR* mutation	Prior treatment, duration	Specimens	Genes tested
Ushiki et al. [7]	Adenocarcinoma, ex19 del	Gefitinib, 3 months	Autopsy	*EGFR*
Toda‐Ishii et al. [8]	Adenocarcinoma, L858R	Gefitinib, ND	Biopsy (femur met)	5 genes (direct sequencing)
Masuda et al. [9]	Adenocarcinoma, L858R	Erlotinib, 12 days	Autopsy	*EGFR* L858R (IHC)
Nishimatsu et al. [10]	Adenocarcinoma, L858R	Gefitinib, 3 months Erlotinib, 6 months	Biopsy (bronchoscopy)	*EGFR* L858R (IHC)
Nagai et al. [11]	Adenocarcinoma, L858R	Osimertinib, 46 months	Surgical resection	46 genes (ODxTT)
Ours (this case)	Adenocarcinoma, ex19 del	Osimertinib, 3 months Interruption, 3 months Gefitinib, 5 days	Autopsy	634 genes (CGP)

Abbreviations: CGP, comprehensive genome profiling; IHC, immunohistochemistry; met, metastasis; ND, not described; ODxTT, oncomine dx target test.

Regarding the genomic findings in this case, first, two studies reported *EGFR*‐activating mutations in approximately 20% of pulmonary pleomorphic carcinoma [18, 19]. Recently, Nagano et al. reported that *TP53* (71%), *KRAS* (27%), *PTPRD* (22%), *ARID2* (14%), *NF1* (12%), *EGFR* (8%), *HRAS* (4%), *MET* exon 14 skipping (4%), *MAP2K1* (4%), *PIK3CA* (4%), *EML4*‐*ALK* fusion (2%), *NRAS* (2%) and *BRAF* (2%) alterations were detected among 52 patients with pulmonary PC [20]. Most recently, Pang et al. found alterations in cancer‐critical genes such as *TP53*, *NF1*, *PIK3CA* and *RB1* in re‐biopsy tumor samples of sarcomatoid transformation from six *EGFR*‐altered lung adenocarcinomas while *KRAS* mutations were only found in primary sarcomatoid carcinomas [21]. *NF1*, one of the mutated genes in this case, is a tumor suppressor gene which functions as a negative regulator of the Ras pathway, and its loss of function mutations are found in pleomorphic sarcomas [22, 23]. Considering these facts, the gene alteration profile of *BRAF* G466A, *KRAS* L19F and *NF1* Q2324* mutations in addition to *EGFR* E746_A750del might be associated with the pleomorphic histology in this case.

## 4 Conclusion

We experienced a case of *EGFR*‐mutated, transformed PC, in which we identified accompanying gene alterations via comprehensive genomic profiling. Anti‐cancer treatments with radiation and EGFR‐TKIs and the gene alterations might induce the histological transformation and resistance to EGFR‐TKIs. This report provides insight into the involvement of genetic events in PC transformation in *EGFR*‐mutated lung cancer.

## Author Contributions

**Yuki Akazawa:** investigation, writing – original draft, visualization, resources. **Takashi Sato:** conceptualization, methodology, investigation, funding acquisition, writing – original draft, writing – review and editing, project administration, resources. **Yuri Yagami:** writing – review and editing, investigation, resources, visualization. **Yurika Kesen:** writing – review and editing, investigation, resources, visualization. **Tomohiro Furo:** writing – review and editing, resources. **Atsuko Watanabe:** writing – review and editing, resources. **Jumpei Endo:** resources, writing – review and editing. **Maito Nakano:** writing – review and editing, resources. **Hiroto Kurata:** resources, writing – review and editing. **Hiromi Matsuo:** writing – review and editing, resources. **Yoshiro Nakahara:** writing – review and editing, resources. **Jiichiro Sasaki:** supervision, writing – review and editing. **Katsuhiko Naoki:** writing – review and editing, resources, supervision.

## Funding

This work was supported by Japan Society for the Promotion of Science (23K07609).

## Ethics Statement

Written informed consent was obtained from the family of the deceased patient. The study protocol was approved by the ethical committee of Kitasato University Hospital (approval number G21‐10).

## Conflicts of Interest

T.S. reports honoraria from Chugai Pharma, Bristol Myers Squibb, AstraZeneca, Ono Pharmaceutical, Nippon Kayaku, Eli Lilly, Boehringer Ingelheim, Daiichi‐Sankyo, Takeda Pharmaceutical, MSD, and Asahi Kasei Pharma. Y.N. reports honoraria from Takeda Pharmaceutical, Eli Lilly, Kyowa Kirin, Boehringer Ingelheim, AstraZeneca, and Bristol Myers Squibb. J.S. reports honoraria from AstraZeneca, EP‐Link, A2 Healthcare, MSD, Ono Pharmaceutical, Kyorin Pharmaceutical, Kyowa Kirin, Daiichi‐Sankyo, Taiho Pharmaceutical, Chugai Pharma, Terumo, Eli Lilly, Nippon Kayaku, Becton Dickinson, Novartis, Bayer, Bristol Myers Squibb, and Yakult. K.N. reports honoraria from Chugai Pharma and AstraZeneca, and grants/contracts from Boehringer Ingelheim, Ono Pharmaceutical, Chugai Pharma, Taiho Pharmaceutical, and Parexel International.

## Data Availability

The data that support the findings of this study are available from the corresponding author upon reasonable request.
